# Impact of personal economic environment and personality factors on individual financial decision making

**DOI:** 10.3389/fpsyg.2014.00158

**Published:** 2014-03-04

**Authors:** Susanne Prinz, Gerhard Gründer, Ralf D. Hilgers, Oliver Holtemöller, Ingo Vernaleken

**Affiliations:** ^1^Department of Psychiatry, Psychotherapy and Psychosomatics, Centre for Integrative Psychiatry, University Hospital of Psychiatry ZurichRheinau, Switzerland; ^2^Department of Psychiatry, Psychotherapy and Psychosomatics, Faculty of Medicine, RWTH Aachen UniversityAachen, Germany; ^3^Institute of Medical Statistics, Faculty of Medicine, RWTH Aachen UniversityAachen, Germany; ^4^Department of Macroeconomics, Halle Institute for Economic ResearchHalle, Germany; ^5^Martin Luther UniversityHalle, Germany

**Keywords:** neuroeconomics, decision making, personality, environment, risk aversion investment, expected utility

## Abstract

This study on healthy young male students aimed to enlighten the associations between an individual’s financial decision making and surrogate makers for environmental factors covering long-term financial socialization, the current financial security/responsibility, and the personal affinity to financial affairs as represented by parental income, funding situation, and field of study. A group of 150 male young healthy students underwent two versions of the Holt and Laury (2002) lottery paradigm (matrix and random sequential version). Their financial decision was mainly driven by the factor “source of funding”: students with strict performance control (grants, scholarships) had much higher rates of relative risk aversion (RRA) than subjects with support from family (ΔRRA = 0.22; *p* = 0.018). Personality scores only modestly affected the outcome. In an ANOVA, however, also the intelligence quotient significantly and relevantly contributed to the explanation of variance; the effects of parental income and the personality factors “agreeableness” and “openness” showed moderate to modest – but significant – effects. These findings suggest that environmental factors more than personality factors affect risk aversion.

## INTRODUCTION

In economics and politics, it is generally assumed that individual financial decision making is a cognitive process based on rational weighing of the corresponding gains, losses, and risks. This very mechanistic approach culminated very early in Pascal’s Wager (1670). Pascal (1670) proposed that it might be logical to believe in God when considering the risks of the false negative expectation (hell) and the gain in the correct positive case (paradise) against the minimal effects of a correct denial of God. More generally, Pascal (1670) argued that a decision maker should assess the desirability of each option by representing it as having a value and a likelihood. This early concept was to some extent adapted to the circumstances of human behavior by Bernoulli (1738) who said that the gain/risk relationship is not linearly related due to a general risk avoidant factor. Thus, a logarithmic transformation of value has come to be known as a “utility,” and the sum of probability-weighted utilities has come to be known as “expected utility” (Neumann and Morgenstern, 1944). This and consecutive models, however, are highly mechanistic and reductionistic. Individual differences in the weighing of risks and gains, the subject’s level of risk aversion or reward seeking, and the complex neurobiological process of decision making are not integrated in these models.

Over the past years, neurobiologists have shown increasing interest in neuroeconomic issues such as the impact of risk seeking or risk aversion. Neuroeconomics as a new field of interdisciplinary research is currently on the rise, analyzing the relationship between economic theories and neuroscientific considerations (Camerer et al., 2004, 2005; McFadden, 2005; Camerer, 2007). Although the importance of individual behavioral factors and neuropsychological issues in economic decision making is now increasingly recognized, neuroeconomic issues still do not receive adequate attention and individual factors that influence risky decision making are not always taken into consideration in socioeconomics. In fact, cerebral activation studies revealed findings, which suggest a complex processing of gain and loss expectations which could roughly be separated into two pathways: fluorodeoxyglucose positron emission tomography (FDG-PET) and functional magnetic resonance imaging (fMRI) studies uncovered relevant structures and cerebral networks which are active prior to risky choices (in particular nucleus accumbens, NAc) or prior to loss prediction (e.g., anterior insula), suggesting that a suitable balance of gain/loss prediction is important for adequate decision making (Knutson et al., 2008; Balodis et al., 2012). Paradigms that show the associations between gain/loss prediction and spatially selective brain activations primarily mimic short-term financial decisions.

Individuals may show different long-term patterns of decision making including general traits such as risk aversion, reward seeking, or the individual levels of liberality and velocity in decision making. Human behavior may be regarded as the sum of a person’s decisions and contributes essentially to the formation of an individual’s personality. Thus, it is not surprising that some previous studies using personality inventories reported some positive correlations between “novelty seeking” items and risk-loving behavior dimensions (Kelley et al., 2004). “Impulsivity” was also found to be a contributing factor (Burnett Heyes et al., 2012). These investigations have not been restricted to financial decision making, but include general risk seeking behavior. It appears to be likely that personality traits have a relevant impact on financial decisions. It is, furthermore, noteworthy that much of the previous literature was performed by using gambling tasks, which, however, do not fully depict the processes of most of the financial decisions. The Holt and Laury paradigm was claimed to be more a financial decision task under situations of uncertainty rather than a gambling task (Deck et al., 2008); thus, it better satisfies the needs of the present experiment.

Nevertheless, a model including gains, losses, risks, and individual patterns of decision making (independent of the etiopathological hypotheses) still appears to be too reductionist. In general, human decisions also depend on environmental conditions, on individual experiences and preoccupations; also age and gender were previously shown to be important influencing factors (e.g., Harris et al., 2006). In particular the present personal economic environment (security, independence, wealth) as well as the history of economic experiences may have a reasonable influence on financial decision making. Furthermore, individual economic insight and preoccupations may bias financial decisions that way that they are not simply an analog of the subject’s overall risk seeking and avoidance.

The present experimental and hypothesis-driven investigation was designed to prospectively test the relative influence of economic environmental and personality factors on financial decision making. It was focused on long-term financial socialization/experiences, the current financial security/independence/responsibility, and the personal affinity to financial affairs; these environmental factors were characterized by few, clearly defined, objective, and relevant surrogate parameters. (parental income, funding situation, and field of study). On the other hand, already known important and influencing parameters such as age, gender, and cognitive performance have been highly restricted. Also the status of career and the educational level was highly restricted (students) in order to reduce the complexity of influencing factors. The subject’s individual risk aversion was quantified using a well-established lottery paradigm in its original version with all risk options uncovered (matrix version) and in a rearranged less transparent version (Holt and Laury, 2002). We hypothesize that environmental factors effectively influence the decision making process and exhibit interactions with personality factors.

## MATERIALS AND METHODS

### SUBJECTS

The study was approved by the local Ethics Committee. For recruitment, flyers and advertisements were displayed in lecture halls and university buildings. In order to avoid unintended variance and confounding factors, narrow inclusion criteria were employed: all subjects had to be male, mentally healthy (including absence of substance abuse and dependency), free of relevant somatic complaints and aged between 18 and 25 years. 150 subjects (age: 23.7 ± 3.4 years [mean ± SD]) were included for participation after a telephone or email based pre-screening procedure. They were matriculated predominantly for studies in medicine (*n* = 41), economics (*n* = 66), or teacher training (*n* = 20). Further exclusion criteria comprised mental diseases in first-degree relatives, psychotropic drug intake in the last 6 months, and any chronic treatment with potentially psychoactive drugs.

### STUDY DESIGN

The subjects were scheduled for participation in groups of 16–44 persons. All subjects sat in front of a computer terminal and found additional working materials on their desk. As surrogate parameters for the influence of environmental long-term financial socialization, the current financial security/responsibility, and the personal affinity to financial affairs the respective parameters were surveyed: parental income, funding situation, and field of study. Thus, the subjects were asked to provide information about their age, their field of study, the estimated income of their parents [(1): up to 1,500 €/month; (2): 1,500–3,500 €//month; (3): 3,500–7,000 €/month; (4): more than 7,000 €/month], and the major source for the means of subsistence [(1): loan; (2): grant/scholarship; (3): parents/family; (4): own income; (5): other]. Subjects were free to refuse personal information about their parents’ income (*n* = 19) and their funding situation (*n* = 2). For further analyses, the source of funding groups 3 and 4 were merged in the “Family/Work” group due to the fact, that these students usually do not experience a high intensity of performance control; conversely, groups 1, 2 (with high performance control) and 5 were merged. Personality dimensions were acquired by use of the NEO Five-Factor Inventory (NEO FFI; Costa and McCrae, 1992); the subject’s intelligence was estimated using the culture fair intelligence test (CFT; Weiß, 2006). Finally, the subject performed the original and a modified version of the Holt and Laury lottery paradigm (Holt and Laury, 2002). All gains which were individually achieved (see below) were paid off directly, ramped up by a small fixed amount of 10€ which served as a blanket reimbursement for time and travel expenses. All personalized information including the amount of the individual payoff was pseudonymized using a unique ID number.

### CULTURE FAIR INTELLIGENCE TEST (CFT 20-R) – SHORT FORM

This task estimates the individual ability to uncover logical relations, series and classifications by minimizing the bias of language and cultural background (Weiß, 2006). There are only short instructions (10 min). It consists of four subtests using structured images indicating (1) series [15 items], (2) classifications [15 items], (3) matrices [15 items], and (4) topologies [11 items]. For the short version of the CFT 20-R, the subject has a maximum of 27 min for solving the whole task (7 + 7 + 6 + 7 min). For the respective age group (20–25 years.), the CFT-reference sample showed an average number of correctly solved items of 39.5 out of 56.

### NEO FIVE-FACTOR INVENTORY

This multidimensional NEO FFI consists of 60 items partitioned into five dimensions (N = *neuroticism*, E = *extraversion*, O = *openness to experience*, A = *agreeableness*, and C = *conscientiousness*), known as the Big Five, consisting of 12 items each (Costa and McCrae, 1992), ranging from “strongly agree” to “strongly disagree” (coded in five values). In this study, we used the German version of the NEO FFI (Borkenau and Ostendorf, 2008).

### LOTTERY PARADIGM

**FIGURE 1:**
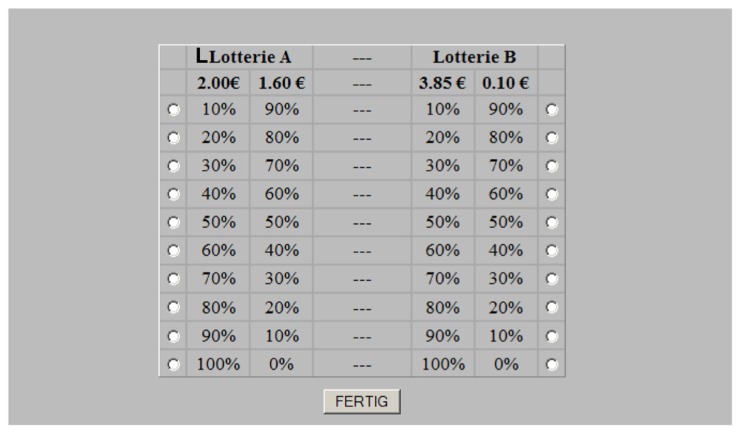
**Screenshot of the matrix version in which all the iterations were presented simultaneously.** In the sequential version (not displayed here), only one iteration was presented at a time.

Basically, we determined the level at which an individual is willing to surrender a comparatively safe combination of stakes in favor of a more risky combination of stakes in a lottery paradigm. To this end, we used the same experimental setup as Holt and Laury (2002) did in their well-established real-payment driven lottery experiment. The lottery was played on computers. In short, the Holt and Laury paradigm presents to the subjects a matrix of ten subsequent lottery draws. For each of the draws, the subject can choose between a low-risk and a high-risk option. For the low-risk options, there is only a slight difference between the respective high payoff (2.00€) and the respective low payoff (1.60€). In the high-risk option the respective payoffs are 3.85€ vs. 0.10€. The ten subsequent draws differ from each other by increasing probabilities of the high payoff, starting with *p *= 0.1 and ending at *p *= 1.0 (nine increments of 0.1). All of the ten lotteries are clearly comprehensible and simultaneously presented in one display. After a time of reflection the subject is asked to choose between the high-risk and low-risk options for all draws. Usually, the subjects begin with the low-risk option at *p *= 0.1 and switch to the high-risk option later. In the present study, the whole procedure was repeated with payoffs that were multiplied by a scaling factor [high-stake condition (HSS)]. This scaling factor (*s*) was fixed to a standard value of *s *= 20. Finally, a third lottery was conducted again using a scaling factor of 1; the latter was, however, not included in the calculation of the relative risk aversion (*r*, RRA) and the wealth factor (*W*). Before the lottery paradigm was performed in the original (matrix) version (for an example screenshot see **Figure 1**), a modified version was played. In order to elicit more spontaneous and less deliberated choices, the lottery was played in a sequential version. The general conditions of the probabilities, stakes, and gains remained unchanged. The player, however, was presented only one lottery iteration per screen. These single draws were presented in random order with respect to the winning probability (*p*). Each probability level was presented three times so that in each stake condition (high-stake vs. low-stake) the subject had to make 30 decisions. In the matrix version, the earnings of the low-stake condition (LSS) were displayed immediately after the game but needed to be waived before entering the HSS. The earnings of the HSS were again displayed before entering the second LSS (only in the matrix version). The earnings of the second low-stake lottery (matrix version) were added to the high-stake lottery payoff. The primary outcome parameters reflect the level of probability at which the subject switches from the relatively safe to the risky options. In order to determine this level unequivocally, within one matrix only one switch (to the riskier option) was allowed. Otherwise the subject’s choices were considered inconsistent and the corresponding observations were dropped from the statistical analyses. Consequently, in the original matrix version, there are three switching points: one for the first low-stake, one for the second low-stake and one for the HSS (LSM1, LSM2, and HSM). These primary outcome parameters can be inserted in the calculation of the individual’s RRA and his wealth factor – for details see Holt and Laury (2002) and Heinemann (2008). In short, the basic associations between the expected utility of the high-stake and the expected utility of the LSS (when unaffected by the stake condition) were defined as *p*(2*s*)^1^^-^**^r^ + (1-*p*) × (1.6*s*)^1^^-^**^r^ = *p*(3.85*s*)^1^^-^**^r^ + (1-*p*) × (0.1*s*)^1^^-^**^r^ where *p* is the probability at which the subject switches, *s* is the scaling factor (20), and *r* is the RRA. The values “2,” “1.6,” “3.85,” and “0.1” reflect the before-mentioned payoffs in Euro. Increasing dissociations of the observed *p* between the low-stake and HSSs would then reflect increasing RRA. Another factor, which can theoretically affect the switching level is the initial wealth (*W*). Inclusion of this factor in the above mentioned equation as offset for the expected gains yields: *p*(*W* + 2*s*)^1^^-^**^r^ + (1-*p*) × (*W* + 1.6*s*)^1^^-^**^r^ = *p*(*W* + 3.85*s*)^1^^-^**^r^ + (1-*p*) × (*W* + 0.1*s*)^1^^-^**^r^. Estimates of RRA and *W* were calculated after insertion of the observed switching probabilities in the first low-stake and the HSS (p^*^_L_ and p^*^_H_).

Since in the sequential paradigm inconsistencies regarding switching back were not only allowed but intended, these subjects were not excluded; the switching point was calculated by means of a curve-fitting procedure. To this end, for each decision the respective winning probability (*x*) was plotted against a value of “1” (high-risk choice) or “-1” (low-risk choice) (*y*). That point where the fitted curve crossed the *x*-axis was defined as the switching point (LSS for the LSS and HSS for the HSS).

### STATISTICS

Since we were interested in a monotone relationship between the outcome parameters HSS, LSS, *W*, RRA we calculated Spearman rank correlation coefficients with corresponding 95% confidence intervals. Group comparisons were carried out by *t*-tests. Test results were considered significant, if the *p*-value is below the significance level of 5%. We furthermore fitted an analysis of variance model (ANOVA, mixed model; SAS proc mixed, SAS Corp., Cary, NC, USA) to the results of RRA including the explanatory continuous variables FFI-N, FFI-E, FFI-O, FFI-A, FFI-C, and IQ_(CFT) and categorial variables “field of study,” “parental income,” “family support/work” (FS/W). Initially, the two-way interactions between the FFI variables and IQ_(CFT), “field of study,” “parental income,” and “family support/work” as well as all two-way interactions between “field of study,” “parental income,” and “family support/work” were included in the model. We used a backward selection procedure in order to find the model, which would consist of significant factors (*p* < 0.05), only. Furthermore, we applied a check for parameter instability and restricted maximum likelihood effects. We crosschecked the model with the model in which only main effects were used as model building factors. Due to high overlap between these models, the main-effect-only results are reported. To describe the effects, we used linear regression as well as linear contrast for the categorial variables.

## RESULTS

### DESCRIPTIVE STATISTICS

The students who participated in this study showed a mean RRA ± SD of 0.51 ± 0.56 and a wealth factor (*W*) of 0.75 ± 1.9 (matrix version). In the sequential paradigm, the calculated point of equal likelihood for risky/safe decisions was 0.56 ± 0.22 in the LSS and 0.61 ± 0.25 in the HSS. For description of the funding situation see **Table 1**; two students refused to respond. With respect to parental income, 12 students (8.0%) reported very low-income conditions whereas 15 subjects (10.0%) had parents with an estimated monthly income of more than 7,000€. The culture free intelligence quotient (IQ) was 114.5 ± 14.3.

**Table 1:** Demographics and basic outcome parameters.

	All *n* = 150 (100%)	Students of economics *n* = 66 (44.0%)	Students of other fields of study *n* = 61 (40.7%)	n/a *n* = 23 (15.3%)
Age	23.7 (±3.4)	24.2 (±4.4)	23.1 (±2.4)	24.0 (±2.6)
Parental income				
<1,500 €/m	12 (8.0%)	9 (13.6%)	1 (1.6%)	2 (8.7%)
1,500–3,500 €/m	45 (30.0%)	20 (30.3%)	18 (29.5%)	7 (30.4%)
3,500–7,000 €/m	59 (39.3%)	22 (33.3%)	26 (42.6%)	11 (47.8%)
>7,000 €/m	15 (10.0%)	5 (7.6%)	8 (13.1%)	2 (8.7%)
n/a	19 (12.7%)	10 (15.2%)	8 (13.1%)	1 (4.3%)
Funding				
*BAFoeG*/scholarship	29 (19.3%)	16 (24.2%)	10 (16.4%)	3 (13%)
Family	87 (58.0%)	37 (56.1%)	40 (65.6%)	10 (43.5%)
Work	30 (20.0%)	11 (16.7%)	9 (14.8%)	10 (43.5%)
n/a	4 (2.6 %)	1 (1.5%)	2 (3.3%)	–
IQ [CFT]	114.5 (±14.3)	111.7 (±15.4)	116.6 (±13.8)	116.6 (±11.2)
NEO FFI				
Neuroticism	17.4 (±6.9)	17.8 (±7.5)	17.8 (±6.0)	15.4 (±7.4)
Extraversion	29.8 (±5.7)	29.9 (±6.0)	29.4 (±5.0)	30.7 (±6.8)
Openness to experience	28.3 (±6.0)	28.5 (±5.9)	27.0 (±5.8)	31.1 (±5.8)
Agreeableness	28.6 (±5.4)	29.5 (±5.4)	27.3 (±5.5)	29.3 (±4.9)
Conscientiousness	28.6 (±6.4)	29.9 (±6.6)	28.2 (±5.7)	33.0 (±6.6)
Lottery – matrix version				
RRA	0.51 (±0.56)	0.49 (±0.66)	0.59 (±0.46)	0.45 (±0.51)
*W*	0.75 (±1.92)	0.94 (±2.65)	0.57 (±1.17)	0.68 (±1.03)
LSM1	0.56 (±0.16)	0.52 (±0.17)	0.60 (±0.15)	0.54 (±0.13)
HSM	0.58 (±0.18)	0.55 (±0.18)	0.60 (±0.18)	0.60 (±0.16)
LSM2	0.56 (±0.16)	0.54 (±0.16)	0.58 (±0.15)	0.54 (±0.18)
Lottery – sequential version				
LSS	0.56 (±0.22)	0.52 (±0.26)	0.63 (±0.16)	0.51 (±0.17)
HSS	0.61 (±0.25)	0.57 (±0.30)	0.65 (±0.21)	0.60 (±0.15)

### TASK COMPARISON

**FIGURE 2:**
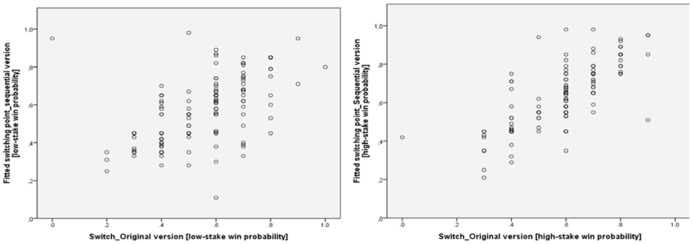
**Graphical comparisons of the switching points (from low to high risk choices) between the original lottery paradigm and the sequential version.** In the original version, which provides the entire information of all lottery draws, no switch-backs were allowed. Thus, three subjects had to be excluded from the matrix game due to inconsistencies in their switch behavior (step back). In six other cases of the high-stake sequential game, no reasonable values of the switching point could be calculated.

Three subjects had to be excluded from the matrix game due to inconsistencies in their switching behavior (switch back). In six cases of the high-stake sequential game no reasonable values of the switching point could be calculated. In the conventional matrix game, the switching level from safe to risky increased from 0.55 ± 0.16 to 0.58 ± 0.17 from the low-stake to the HSSs. This shift toward safer decisions was on trend-level (*p *= 0.063; paired *t*-test). In the sequential version, the difference between HSS and LSS was significant (ΔHSS–LSS: 0.05; *p *= 0.004; paired *t*-test). Nevertheless, the individuals’ decisions in the matrix version correlated highly with those in the sequential version of the game (low-stake: *r *= +0.594; *p *< 0.001; 95% CI: 0.456-0.675/high-stake: *r *= +0.748; *p *< 0.001; 95% CI: 0.666-0.813 Spearman’s correlation; see **Figure 2**). As was expected, the RRA value showed significant correlations with the differences between the switching points of the high-stake and LSSs in the matrix (*r *= +0.359; *p *< 0.001; 95% CI: 0.204–0.496; Spearman’s correlation) and the sequential version (*r *= +0.376; *p *< 0.001; 95% CI: 0.224–0.511; Spearman’s correlation). The following analyses were made using the established RRA and *W* factors of the conventional matrix paradigm.

### ANALYSES OF INFLUENCING FACTORS

**FIGURE 3:**
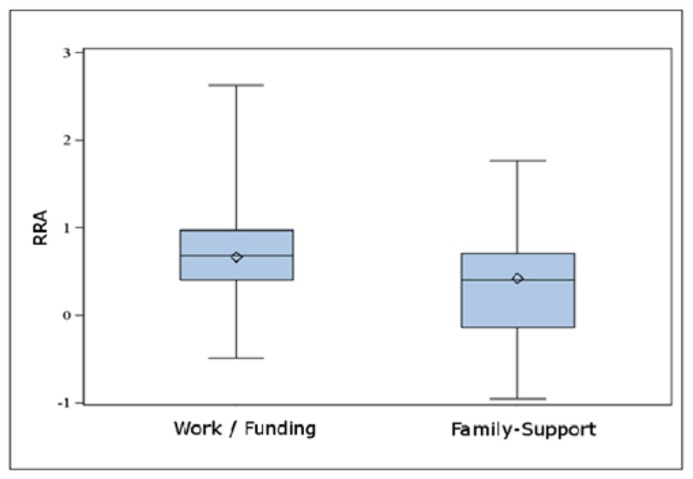
**Depicted are the relative risk aversions (RRA) of students in dependence on their source of funding (support by their families vs. other forms of funding, e.g., own work or grants).** Using the simple unpaired two-tailed *t*-test the difference was statistically significant (ΔRRA = 0.22; 95% CI: 0.04–0.41; *p* = 0.018).

The students’ financial situation had a significant group effect on the RRA. Students who studied under performance control (Loan/BAFoeG) showed much higher risk aversion (ΔRRA = 0.22; 95% CI: 0.04–0.41; *p* = 0.018; unpaired *t*-test; see **Figure 3**). There was no effect on *W*. Parental income, in general, had no influence on RRA and *W*. However, the subgroup with very low parental income (less than 1,500€) showed a tendency to lower RRA values (ΔRRA: 0.32; 95% CI: -0.04–0.67; *p* = 0.084; unpaired *t*-test) compared with the other students. In contrast to our hypothesis, there was no straightforward association between any of the personality dimensions and the RRA or *W* factor. Also, whether a student studied economics had no significant group effect on *W* and RRA. Nevertheless, the field of study had an influence on financial decision making when looking at the basic parameters: students of economics generally switched earlier to the high-risk choice. In the matrix version, this group comparison revealed significant results for the LSS (ΔLSM1 = 0.058; 95% CI: 0.006–0.11: *p* = 0.030; unpaired *t*-test) whereas the HSS showed only trend-level differences (ΔHSM = 0.052; 95% CI: -0.008–0.11: *p* = 0.089; unpaired *t*-test). In the sequential version similar group differences (ΔLSS = 0.077; 95% CI: 0.006–0.15: *p* = 0.033; ΔHSS = 0.061; 95% CI: -0.022–0.14: *p* = 0.147; unpaired *t*-test) could be observed. This difference was much more pronounced when comparing the students of economics to the teacher group (sequential task: ΔLSS = 0.136; 95% CI: 0.016–0.26; *p* = 0.027; ΔHSS = 0.172; 95% CI: 0.026–0.32; *p* = 0.022; matrix task: ΔLSM1 = 0.121; 95% CI: 0.038–0.20; *p* = 0.005; ΔHSM = 0.112; 95% CI: 0.027–0.20; *p* = 0.010; unpaired *t*-test). The RRA was also lower in the group of economic students; the respective differences, however, failed to reach statistical significance. The intelligence (IQ-CFT) did not correlate to any of the task-related outcome parameters in the total sample.

### ANOVA (PROC MIXED, SAS)

**FIGURE 4:**
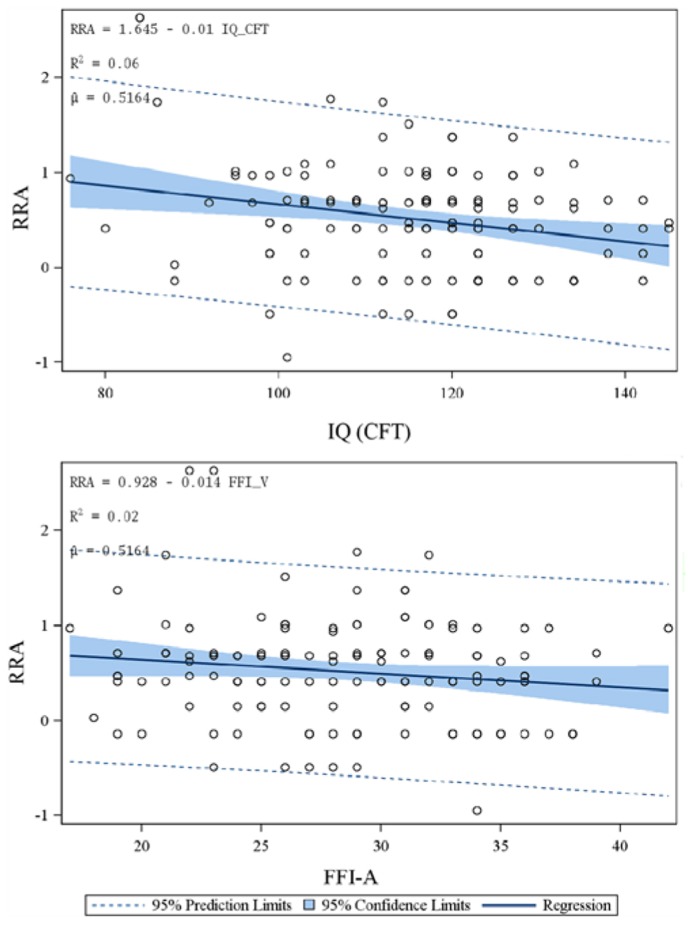
**Simple linear regressions after exclusion of six individual datasets according to the check for parameter instability and restricted maximum likelihood effects (proc-mixed, SAS).** Depicted are individual observations for RRA plotted versus the intelligence quotient measured by the culture free intelligence task (CFT) and the NEO-FFI agreeableness score (FFI-A). Furthermore, the 95% prediction limits (dotted lines) and the 95% confidence limits (blue area) are depicted.

During model fit, the influence diagnostic procedure revealed that the records for subjects number 47, 97, 115, 119 had to be deleted due to parameter instability, and those of subjects number 45, 125 due to high influence on the overall fit (restricted maximum likelihood). We ended with the model exploration analysis, which showed a significant influence of FFI-O, FFI-A, IQ_(CFT), “parental income,” and “family support/work” on the RRA values. **Table 2** displays the results of the mixed model after inclusion of the respective factors. According to this model, in particular the IQ (*R*^2^ = 0.06, *F* = 15.35) and “family support/work” (*F* = 10.40) significantly and relevantly contributed to the explanation of variance. Also, “agreeableness” (*R*^2^ = 0.02, *F* = 7.71) as well as – to a more moderate extent – “openness” (*R*^2^ = 0.01, *F* = 4.52) and “parental income” (*F* = 4.35) significantly added to the explanation of variance of RRA. **Figure 4** shows the individual plots for RRA vs. IQ (CFT) and FFI-A.

**Table 2:** ANOVA results (SAS proc mixed, SAS Corp., Cary, NC, USA) including main effects of two personality factors (NEO-FFI openness/agreeableness), IQ (CFT), “Parental Income” (ParIncome), and Family Support/Work (FS/W) on relative risk aversion (RRA).

Effect	DF (num)	DF (den)	*F*	*P*
FFI_O	1	127	4.52	0.0355
FFI_A	1	127	7.71	0.0063
IQ_CFT	1	127	15.35	0.0001
ParIncome	4	127	4.35	0.0025
FS/W	1	127	10.40	0.0016

## DISCUSSION

This investigation was designed to test the influence of intrinsic and environmental factors on the individual’s style of financial decision making. Based on the subsequently described considerations, the protocol of this investigation focused on the Big Five, the parental income, the funding situation, as well as the professional orientation.

(1) The first key finding was a significant association between the current funding situation and the RRA. According to our data, subjects studying under performance control (*BAFoeG*, scholarship) displayed a more conservative attitude toward risk than subjects who received their money from their families or from regular employment. **Figure 3** suggests that this difference is mainly due to the lower RRA values of the students supported by their families.
(2) A second hypothesis could be confirmed: the field of study was significantly related to risk aversion. Apparently, an increased interest in economics and business affairs correlates with a higher probability of making risky decisions. This was particularly striking in the comparison of the economic student group with the teacher group, the latter being highly risk-averse.
(3) A third key finding of the present study was that, contrary to our expectations, personality traits reflected by the Big Five appeared to have only minor and statistically non-significant effects in direct correlational analyses. This was the case for the initially favored item “neuroticism” as well as for any other dimension. The mixed model, however, identified “agreeableness” and “openness” as factors with considerable explanatory power as main effects. Interestingly, subjects with lower “openness” scores exhibited lower risk aversion in their behavior with respect to their financial situation. This association, however, was inversed for the item “agreeableness” (**Figure 4**).
(4) Finally, the results with respect to parental income were not fully conclusive. There was no overall group effect of the latter factor on RRA or *W*; neither was there a correlation. Looking specifically at the subgroup of very low parental income (<1,500€/month), these few subjects (*n* = 10) displayed very low RRA values accompanied by a high variance (mean ± SD: 0.22 ± 0.60).

Taken together, the current source of funding had the highest influence on the assessment of risk in financial decisions; intelligence, however, was also significantly modulating the risk strategy, although we had expected that the variance of this important factor would be minor, since only successful students were included. The descriptive statistics, nevertheless, showed that the SD was nearby 15, thus suggesting a common variation, which is merely shifted to higher CFT values. Since the matrix version of the Holt and Laury paradigm depends on a reflection of expected wins, low RRA values might be partly explained by the lack of risk recognition. Cokely and Kelley (2009) reported the relevance of cognitive abilities on risky choice decisions. Although in the present study cognitive measures are restricted to the CFT, the unexpected high variance of CFT-IQ in the present group causes a congruent effect on RRA.

In the present study the item “parental income” as well as the item “source of funding” were thought to reflect the individual’s relationship to money, assuming lower RRA in students with substantial support by their families and a history of financial security. Including only a homogenous group of participants enabled us to uncover the impact of targeted influencing factors such as money-related long-term socialization/experiences (parental income), current financial security/independence/responsibility (funding situation), and the personal affinity to financial affairs (field of study). The prospective design and the laboratory conditions revealed highly reliable outcome parameters in respect to financial risk aversion but reduce the maximum number of participants. This urged to reduce the number of outcome variables and demanded a homogenous study group. As mentioned before, there are many possible influencing environmental factors (e.g., age, career process, misfortune, health, family situation). The present investigation, however, wants to focus on the impact of present and past financial security, independence, and responsibility as well as on the individual proximity to economic topics in comparison to standard personality factors. The restriction of inclusion criteria to male students minimizes the effect of career progress, aging (which causes a shift between conservative to liberal decision bias), heath, and family related problems which causes high variance and complexity but, naturally, decreases the generalizability. The choice of outcome parameters (parental income, funding, field of study) is somewhat arbitrary but appeared to be the best choice to acquire some few easy, objective, relevant data, which highly impact the present and past financial situation.

For quantification of the individual’s risk seeking tendency or risk aversion in financial decisions, the Holt and Laury paradigm (Holt and Laury, 2002) was chosen because the paradigm is well replicated and evaluated; importantly, payoffs comprised of considerable amounts of money (in particular for students). Furthermore, the comparison with gambling tasks (deal-or-no-deal-game) showed that – considering the different associations with the Domain-Specific Risk Taking attitudes – the Holt and Laury paradigm is more specifically depicting the individual tendency for risky financial investments rather than mirroring a general risk seeking (gambling) behavior (Deck et al., 2008). The results of the outcome parameters (LSM1/2, HSM, RRA, and *W*) were on the whole within the range of previous results and analyses (Heinemann, 2008). At the end, an ANOVA showed that personality factors (“openness” and “agreeableness”) as well as “parental income” and “family funding” could significantly explain the variance in financial decision making. Interestingly, also intelligence showed an association with the RRA. In simple group comparisons and correlational analyses, only the funding situation and the parental income showed significant effects. “Field of study” showed group differences in respect to the raw outcome parameters (i.e., LSM1, HSM) but not for RRA.

Doya (2008) mentioned several general issues that influence an individual’s decision: (1) needs/desires, (2) risk/uncertainty, (3) time spent/left for learning, (4) gains vs. losses, (5) cost/efforts, (6) risk/variance, (7) delay discounting, (8) exploration. For most of these confounding variables, the present investigation was strictly controlled. The well described area of stress to avoid losses and to increase gains (Weller et al., 2010) was not the subject of the present study, since for ethical reasons losses were not included in the paradigm. Furthermore, delay discounting (Lagorio and Madden, 2005) and exploration procedures were not in the focus of the protocol. Most importantly, the levels of risk and uncertainty were clearly defined and easy to recognize for the subject. The results, therefore, must be viewed within the scope of expected gains, well-defined risks, the individual’s needs and desires. Against the background of the present results, the tendency to prefer a higher probability to earn a moderate amount of money over a lower probability to earn a greater amount of money is more pronounced in those subjects who need to get by with modest scholarships that are also strictly dependent on the students’ performance. Concluding the experiment with a high chance of gaining the moderate amount of approximately 50 € might represent an attractive prospect for these individuals, as this sum may potentially amount to their weekly living costs. Losing this amount due to the inclination to more risk-loving behavior might be of less importance for subjects with more convenient modes of financing (family) or greater general income (employment). This effect appears to be strictly dependent on the student’s own present personal situation. The parental income showed only a particular significant difference in RRA between students with parents of very low-income and the other students which appears to be counterintuitive and difficult to evaluate due to the small group size (*n* = 10) and the high variance in the present sample. It is noteworthy that this subgroup of subjects also showed significantly lower CFT performance (101.9 ± 17.4 vs. 115.5 ± 13.6; *p* = 0.002, unpaired *t*-test) which might bias the results. For the wealth factor, in fact, a more reasonable group difference could be detected (low-income group: 0.25; other students: 0.73). Since the low-income group also differs in several characteristics from the remaining students (large portion of economics students [75%] and significantly lower intelligence [average IQ = 101]), it is questionable whether the low parental income directly influenced the RRA.

The small effect of the Big Five on risk aversion was somewhat striking. We expected personality to substantially affect gain/risk behavior. Nevertheless, there are only few reports of experiments which correlate personality dimensions to (financial) decision making (Mueller et al., 2010; Wischniewski and Brune, 2012). Some previous investigations have linked the dopamine system – which is highly involved in the decision making process – to personality dimensions. Very early in the history of positron emission tomography (PET) investigations, some associations between the D_2__/3_ receptor availability and personality traits (e.g., personal detachment) were reported (Breier et al., 1998). A recent complex double tracer study showed that a blunted dopamine response under stress conditions was correlated with various subitems of the “neuroticism” dimension. The dopamine driven reward system is the major influence on the processing of gain predictions (Yacubian et al., 2006; Hernandez et al., 2012) whereas loss predictions appear to be processed by different circuits. Nevertheless, “neuroticism” had not the least effect on the RRA results in the present investigation. Two possible reasons may account for this finding. There is a broad spectrum of items that are subsumed in this personality dimension. The items cover a widespread cluster of behavioral traits including such items as depressiveness and anxiety on the one hand and hostility and impulsiveness on the other. We initially suggested that increased impulsiveness might incline the subject toward higher risk seeking behavior; a rather negativistic or anxious attitude, however, might promote choosing safer stakes. Furthermore, the importance of impulsivity might be limited in the Holt and Laury paradigm since the subjects had enough time to make their choice. The directions of the regression analysis for “agreeableness” and “openness,” which unexpectedly reached statistical significance in the ANOVA, were counterintuitive at first glance. However, some previous investigations, which do not focus on financial decision making, are congruent with the present findings. In particular, Gullone and Moore (2000) showed that the “openness” item negatively correlated with reckless risk taking behavior in adolescence whereas “agreeableness” showed positive correlations with thrill seeking behavior. Furthermore, the effect was modest and comparable to the present results.

Another parallel observation was the lack of any association between the “neuroticism” score and thrill seeking or reckless risk taking behavior. We had expected the dimension “neuroticism” to reflect behavioral traits that support anxiety-related as well as impulsive reactions. Whereas there seems to be some association between “neuroticism” and “impulsivity” (Rosler et al., 2004) or compulsive buying behavior (Muller and de Zwaan, 2010) and some studies suggest that anxiety-related traits promote a defensive strategy. On the other hand, anxiety-related traits also seem suited to promote a defensive strategy (Maner and Schmidt, 2006; Giorgetta et al., 2012) Furthermore, a subsample of individuals showing compulsive buying behavior exhibited greatly increased “neuroticism” scores, a report comparing personality traits with the Holt and Laury paradigm as well as with a gambling task mentioned that the respective associations were clearly not congruent; the authors stated that “gambling attitudes do not impact behavior in the Holt and Laury task” (Deck et al., 2008). In a study conducted by Becker et al. (2012), of all personality dimensions “agreeableness” and to a lesser extent “openness” showed moderate correlations with measures of economic preferences. In summary however, they found that the degree of association between personality factors and economic preference was rather low, and since they found no strong linear relationship, they concluded that the two concepts have to be considered as complimentary. They indicate that although there are relationships between them, personality dimensions and individual measures of economic decision making cannot be substituted. So certainly, impulsiveness and economic risk aversion cannot be expected to be found on the same continuum.

Another recent study, again, found that the item “agreeableness” was negatively associated with risk perception in transport behavioral adaptations (Fyhri and Backer-Grondahl, 2012). It is therefore still conceivable that personality factors have a moderate effect on risky financial decision making; however, it is possible that the characterization of personality traits by the NEO FFI is not perfectly suited to describe the underlying mechanisms. It is possible that the use of more diversified inventories differentiating other personality dimensions would have helped to enlighten this relationship.

Another potentially relevant aspect in this discussion is an MRI finding that Tobler et al. (2007) presented. Their data favor the concept that the two basic economic decision parameters, expected value and uncertainty of reward, are coded in distinct key reward structures in the brain, thus confirming that these reward components are processed separately. This aspect has not sufficiently been accounted for in the present study, which might add to the explanation of the rather small association between risk aversion and personality traits. Other studies (Christopoulos et al., 2009; Rudorf et al., 2012) also postulate distinct neural correlates for the different aspects of risky decision making, such as value or magnitude, and expected value, risk and risk aversion, although they also concede that the brain responses they attribute to specific decision parameters are not exclusive, and stress that complex cognitive processes such as decision making involve a complex combination of signals from different brain regions. The key regions involved in risky decision making seem to be the insula, frontal gyrus, anterior cingulate, and ventral striatum.

A recent study investigating the individual determinants of willingness to take risks Dohmen et al. (2011) found a significant influence of age, gender, height, and parental background, confirming on the one hand our decision to control for age and gender as influential factors in order to focus on environmental and personality factors, on the other hand supporting our findings of the relevance of parental background. Interestingly, in the latter study, the authors concluded from their data that the determinants are robust across different contexts of risk taking. Another intention of the present investigation was to clarify the question whether the original version of the Holt and Laury paradigm could be too transparent for the individual’s risk assessment. The sequential version was designed to reduce transparency and provoke more emotional decisions in the subject. The only observable effect in this regard, however, was the frequent occurrence of inconsistencies (switching back) which were explicitly allowed. It was somewhat surprising that the switching points in this approach (sequential version) were, nevertheless, highly correlated with the switching points of the original matrix version and did not provide any further evidence regarding the primary hypotheses. Apparently, decision making without awareness of the whole decision matrix increases the number of inconsistencies, but does not affect the individual’s fundamental risk evaluation.

**FIGURE 5:**
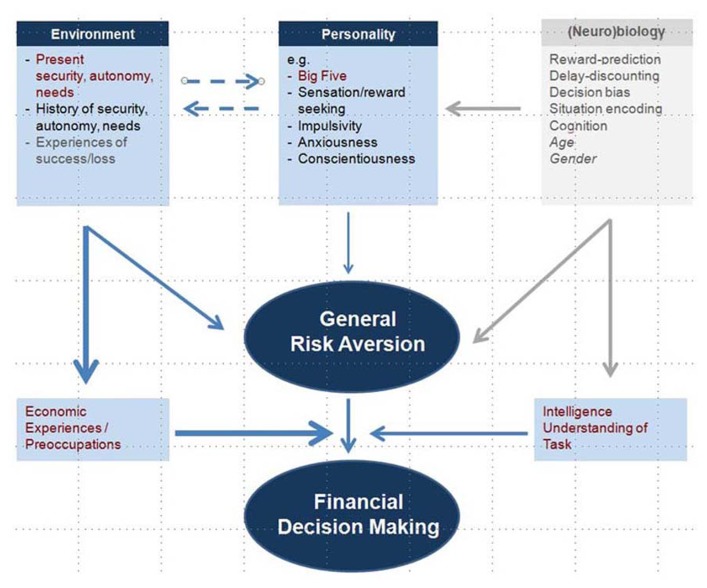
**Model depicting possible influences on risk averse or risk seeking behavior.** Personality factors, as well as neurobiological mechanisms, modulate general risk aversion. However, as enlightened by this study, also environmental factors have a high impact on risk aversion. This may be a direct association, but also mediated by economic experiences. Furthermore, economic preoccupations as well as cognitive performance can independently affect the financial decision making process. (Factors and causalities in gray color depict reasonable associations which were, however, not subject to the present investigation.)

This investigation is an experimental prospective study with a sufficient group size that treats the underlying mechanisms of financial decision making. We claim the sample size of 150 to be sufficient, as the design only included subjects that matched the strictly defined inclusion criteria and who thus represented a relatively homogeneous group. This was done to control for obviously influencing factors such as age, gender, mental illness, and financial difficulties. Taken together, the results strengthen the importance of subject’s current financial environment (security/independence). In the present group, this factor appears to be stronger than the effect of the subject’s (family) history and, surprisingly, than personality factors. Personality to some extent is linked to neurobiological mechanisms, which however, were not subject of the present investigation. A more precise evaluation of certain personality issues and their underlying neurobiology might have revealed stronger associations. As depicted in **Figure 5**, environmental factors might generally modulate the risk aversion/risk seeking behavior but may also act specifically on financial decisions via defined financial experiences. This study, however, also reveals that economic preoccupations and the proximity to economic topics (field of study) are able to modulate the financial RRA independently from the before-mentioned factors. Since economic decisions usually demand a basic understanding of risks, gains and uncertainties, the level of cognitive function is also biasing the decision process. Future investigations should try to simulate loss situations; others might focus on the neurobiological bases of the relationships described above, e.g., the dopaminergic response and neural correlates such as activated networks measured by the MRI.

## AUTHOR CONTRIBUTIONS

Susanne Prinz contributed substantially to the analysis and manuscript preparation. Gerhard Gründer contributed substantially to the conception of the study and the revision of the manuscript. Ralf Hilgers contributed substantially to the analysis of the data and the preparation of the manuscript. Oliver Holtemöller contributed substantially in the conception of the study and the acquisition of data. Ingo Vernaleken contributed substantially to the concept of the study, acquisition of the data, the analysis and the preparation of the manuscript.

## Conflict of Interest Statement

The authors declare that the research was conducted in the absence of any commercial or financial relationships that could be construed as a potential conflict of interest.
